# Supplementary material for: Structural polymorphism and diversity of human segmental duplications
Source: Nat Genet. 2025 Jan 8;57(2):390–401. doi: 10.1038/s41588-024-02051-8 (PMC11821543; doi:10.1038/s41588-024-02051-8)
Supplement: Supplementary file 1 — Supplementary Figs. 1–9 and Supplementary Note. [file 41588_2024_2051_MOESM1_ESM.pdf]

nature genetics

Supplementary information

https://doi.org/10.1038/s41588-024-02051-8

Structural polymorphism and diversity of human segmental duplications

In the format provided by the authors and unedited

SUPPLEMENTARY INFORMATION & FIGURES for Jeong et al.

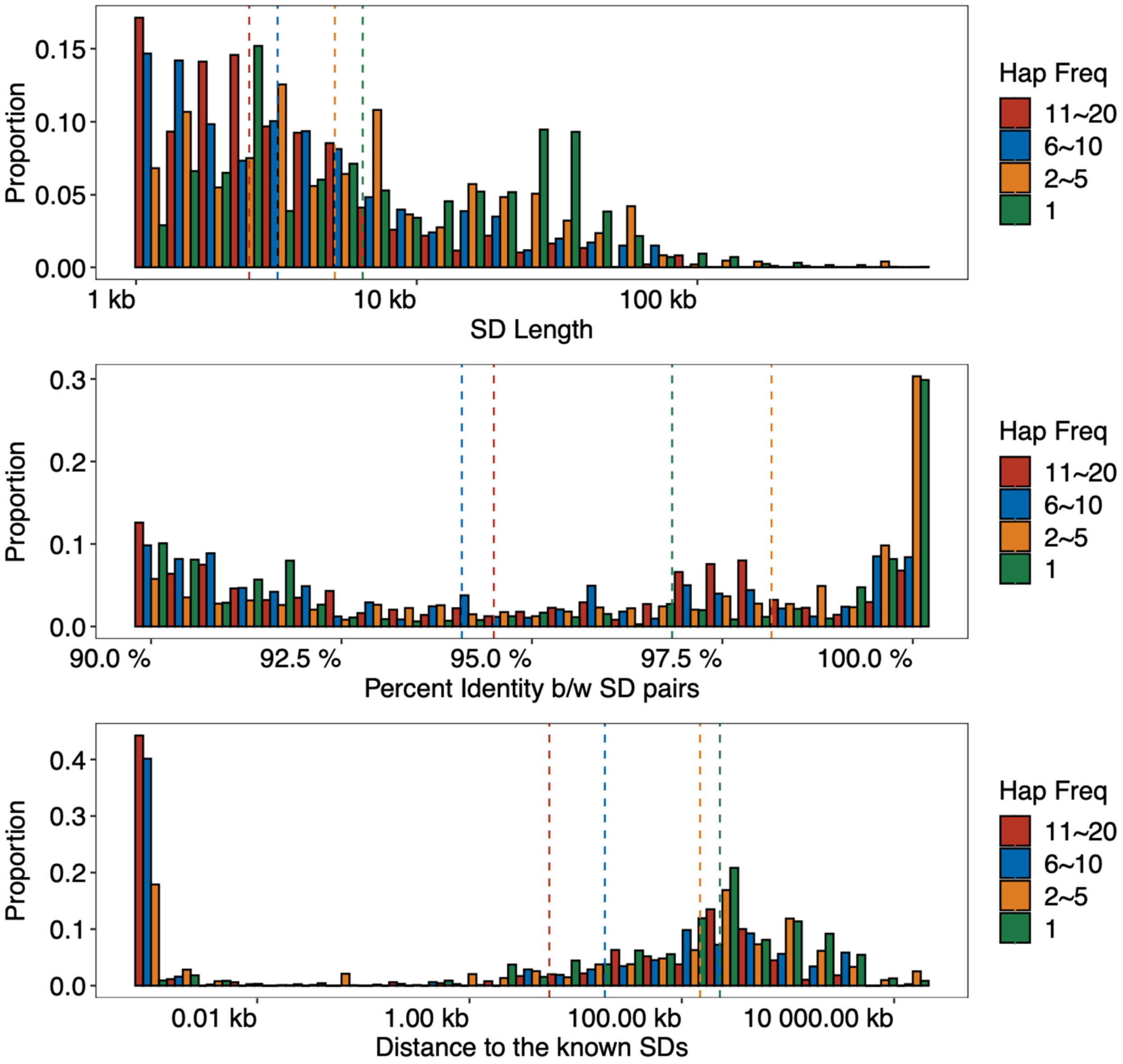

Supplementary Figure 1. Histogram comparing the sequence identity and length of polymorphic segmental duplications (SDs) at different haplotype frequencies.

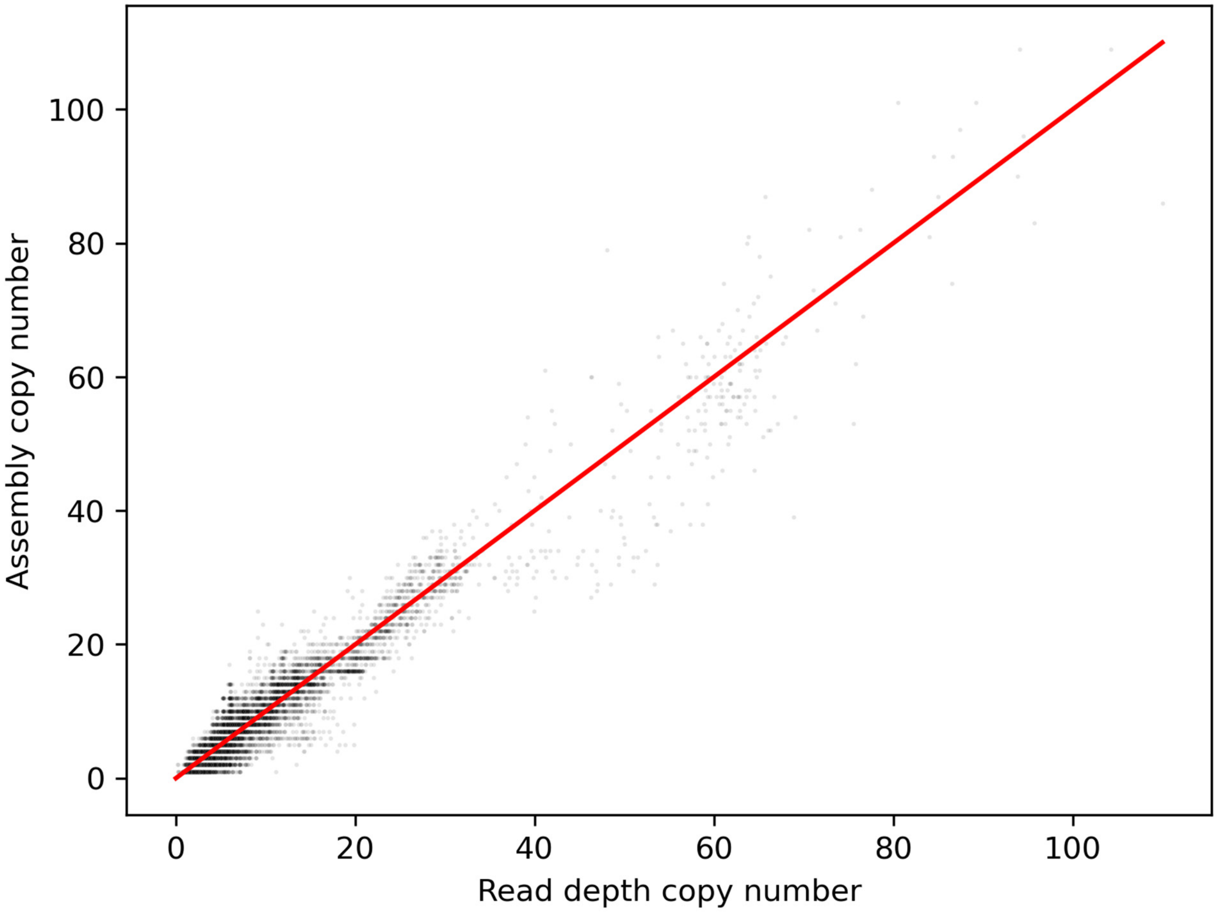

Supplementary Figure 2. Read-depth-based copy numbers estimated with fastCN compared to assembled copy number for each sample, summed between the two haplotypes. Each point represents the copy number estimates for a gene family in a sample (R^2 = 0.94).

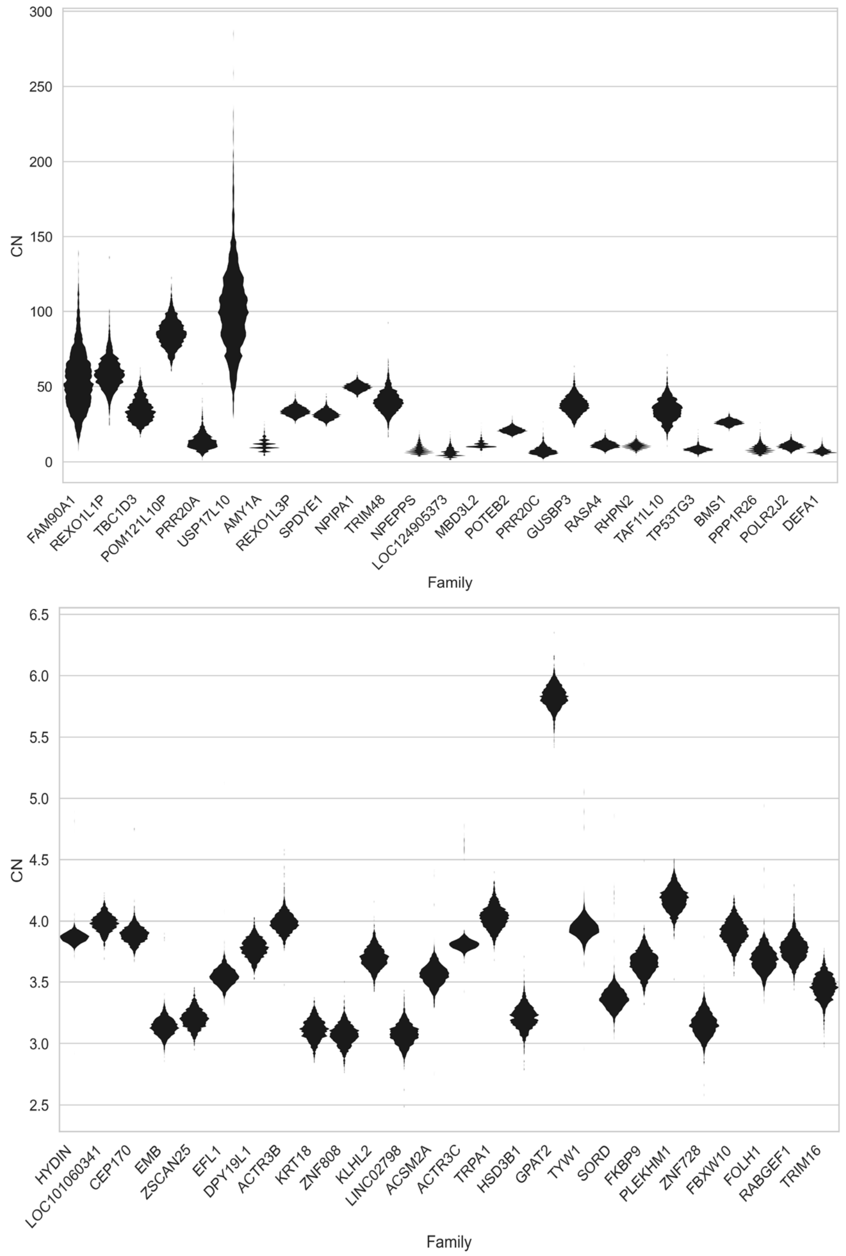

Supplementary Figure 3. Copy number distribution of high- and low-variance gene families. The read-depth copy number of gene families with highly variable (above) and nearly fixed copy number (below) are displayed. Gene families are selected and ordered by variance, requiring an average diploid copy number greater than three.

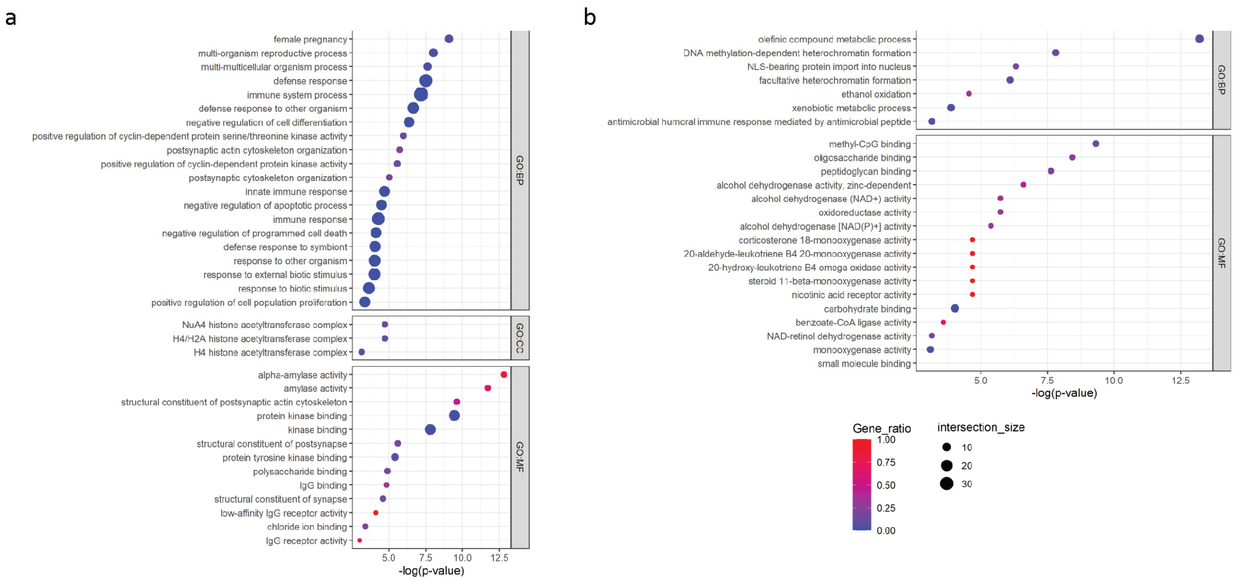

Supplementary Figure 4. Gene ontology enrichment of the (a) top 100 variable gene families (n = 358) and (b) invariable genes (n = 115). The x-axis indicates negative log transformed adjusted p-value. The number of intersecting genes is indicated by the size of the circle and the gene ratio represents the number of intersect/term size. To test statistical enrichment of gene ontology, Fisher's one-tailed test was performed. Multiple test corrections were done by the default g:SCS method of the g:profiler package, accounting for ontologies that are not independent from one another, which is less stringent test than Bonferroni correction or Benjamini-Hochberg FDR.

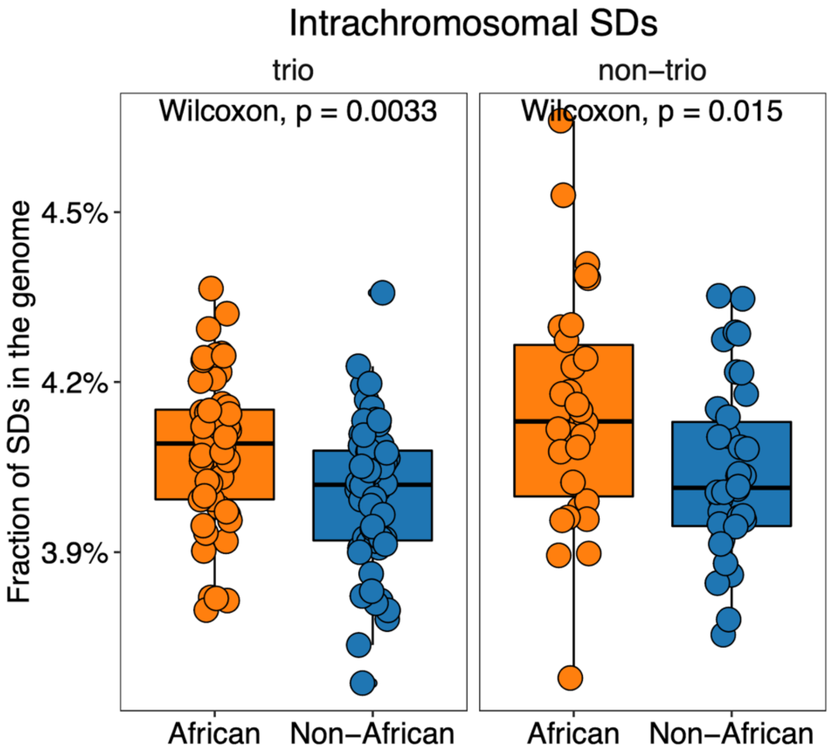

Supplementary Figure 5. Comparison of SD content in trio (n = 102) vs. non-trio (n = 68) genomes. Both datasets show a significant excess of SD content in African samples. The box plot ranges represent the interquartile range (first and third quartile), and the median is indicated by a horizontal line in each box. The whisker indicates the datapoints within 1.5*interquartile range. Two-tailed Wilcox ranked sum test was performed for statistical analyses.

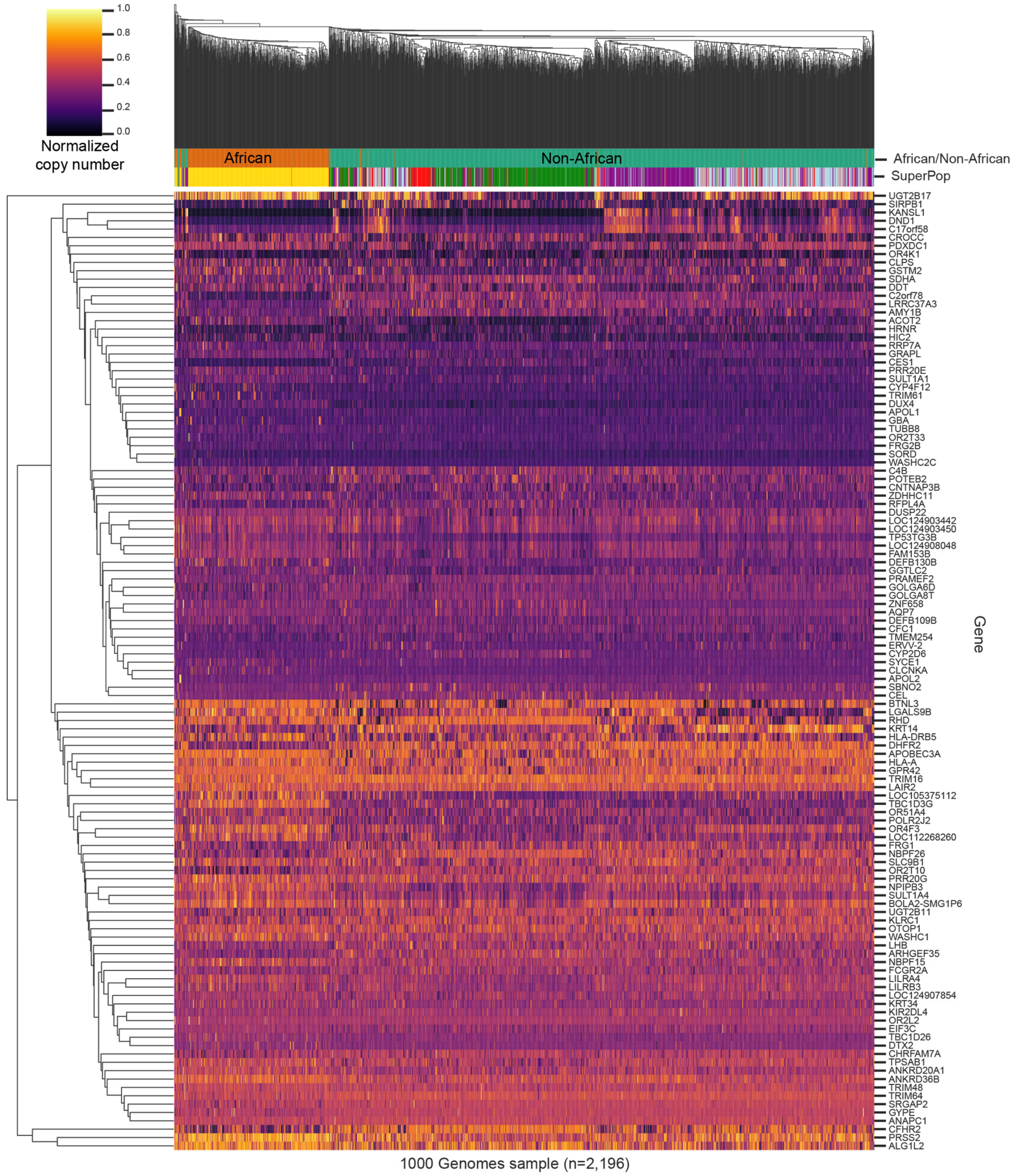

Supplementary Figure 6. Population-stratified genic copy number in 2,196 unrelated individuals from the 1000 Genomes Project. Gene copy number values are centered on the mean for each gene and scaled by unit variance to range from 0-1. One paralog per gene family and duplication block is shown. 73/115 deduplicated population-stratified genes have higher mean copy number in the African group as compared to the non-African group (p=0.002, two-tailed Wilcox ranked sum test). Superpopulations as described in the 1000 Genomes Project are shown above (Africa: gold, East Asia: green, South Asia: purple, Europe: blue, the Americas: red). Copy number estimates by population group (below).

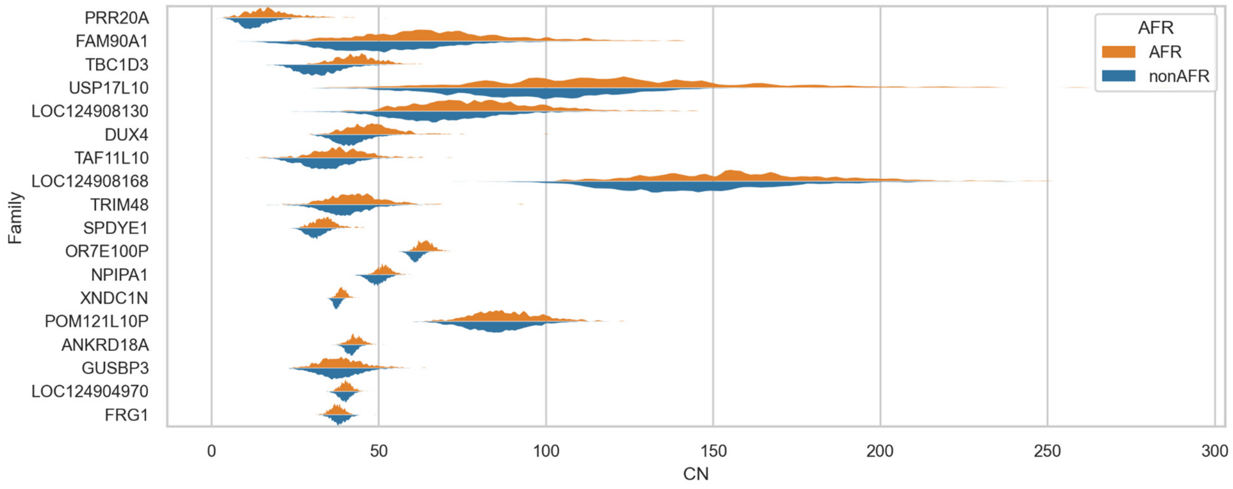

PRR20A
FAM90A1
TBC1D3
USP17L10
LOC124908130
DUX4
TAF11L10
LOC124908168
TRIM48
SPDYE1
OR7E100P
NPIPA1
XNDC1N
POM121L10P
ANKRD18A
GUSBP3
LOC124904970
FRG1
Family
0
50
100
150
200
250
300
CN
AFR
AFR
nonAFR

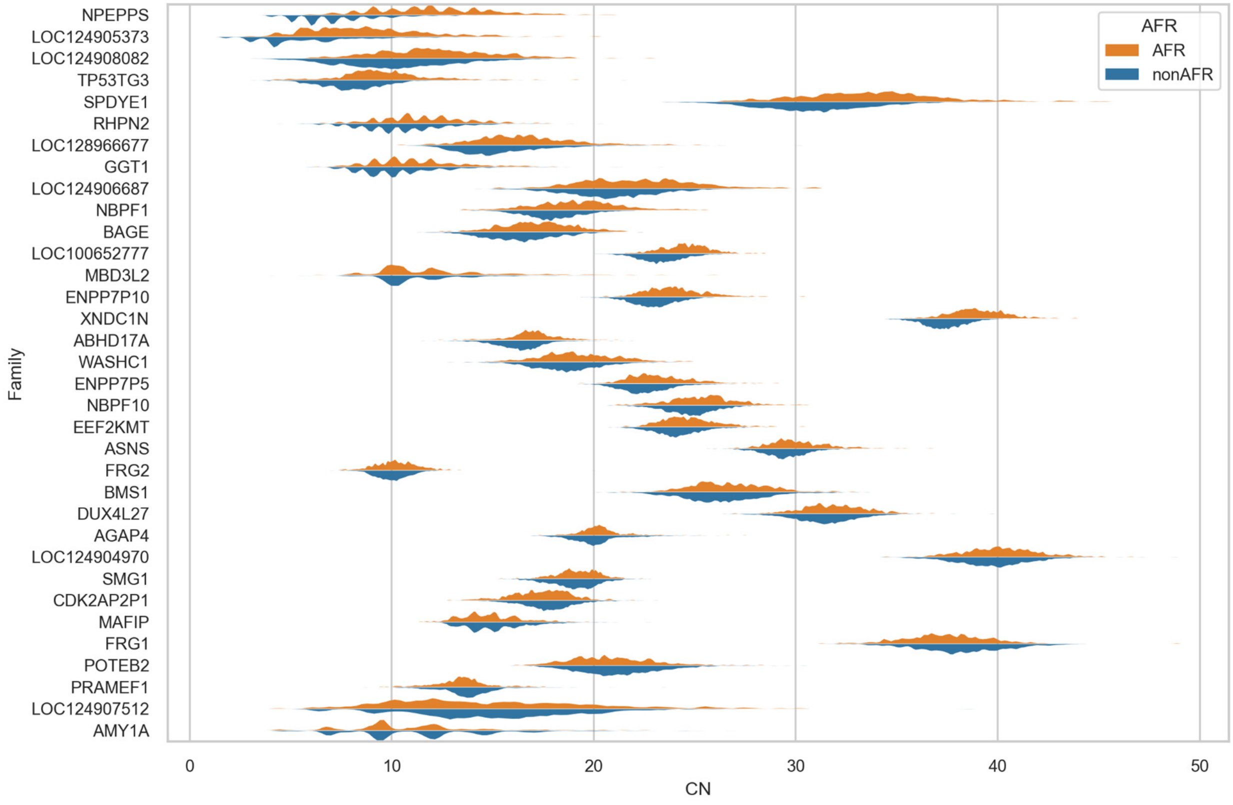

NPEPPS
LOC124905373
LOC124908082
TP53TG3
SPDYE1
RHPN2
LOC128966677
GGT1
LOC124906687
NBPF1
BAGE
LOC100652777
MBD3L2
ENPP7P10
XNDC1N
ABHD17A
WASHC1
ENPP7P5
NBPF10
EEF2KMT
ASNS
FRG2
BMS1
DUX4L27
AGAP4
LOC124904970
SMG1
CDK2AP2P1
MAFIP
FRG1
POTEB2
PRAMEF1
LOC124907512
AMY1A
Family
0
10
20
30
40
50
CN
AFR
AFR
nonAFR

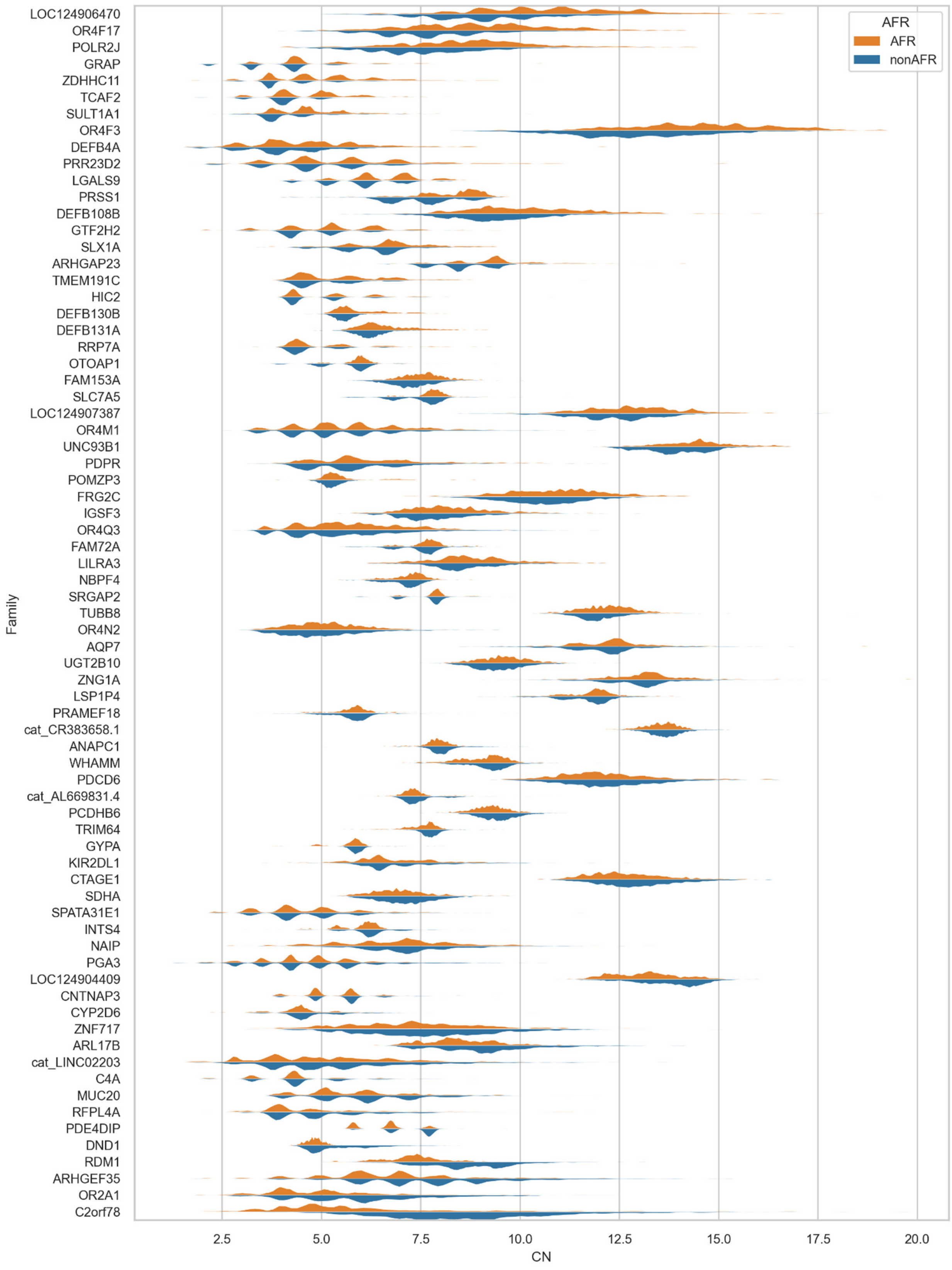
LOC124906470
OR4F17
POLR2J
GRAP
ZDHHC11
TCAF2
SULT1A1
OR4F3
DEFB4A
PRR23D2
LGALS9
PRSS1
DEFB108B
GTF2H2
SLX1A
ARHGAP23
TMEM191C
HIC2
DEFB130B
DEFB131A
RRP7A
OTOAP1
FAM153A
SLC7A5
LOC124907387
OR4M1
UNC93B1
PDPR
POMZP3
FRG2C
IGSF3
OR4Q3
FAM72A
LILRA3
NBPF4
SRGAP2
TUBB8
OR4N2
AQP7
UGT2B10
ZNG1A
LSP1P4
PRAMEF18
cat_CR383658.1
ANAPC1
WHAMM
PDCD6
cat_AL669831.4
PCDHB6
TRIM64
GYPA
KIR2DL1
CTAGE1
SDHA
SPATA31E1
INTS4
NAIP
PGA3
LOC124904409
CNTNAP3
CYP2D6
ZNF717
ARL17B
cat_LINC02203
C4A
MUC20
RFPL4A
PDE4DIP
DND1
RDM1
ARHGEF35
OR2A1
C2orf78
Family
2.5
5.0
7.5
10.0
12.5
15.0
17.5
20.0
CN
AFR
AFR
nonAFR

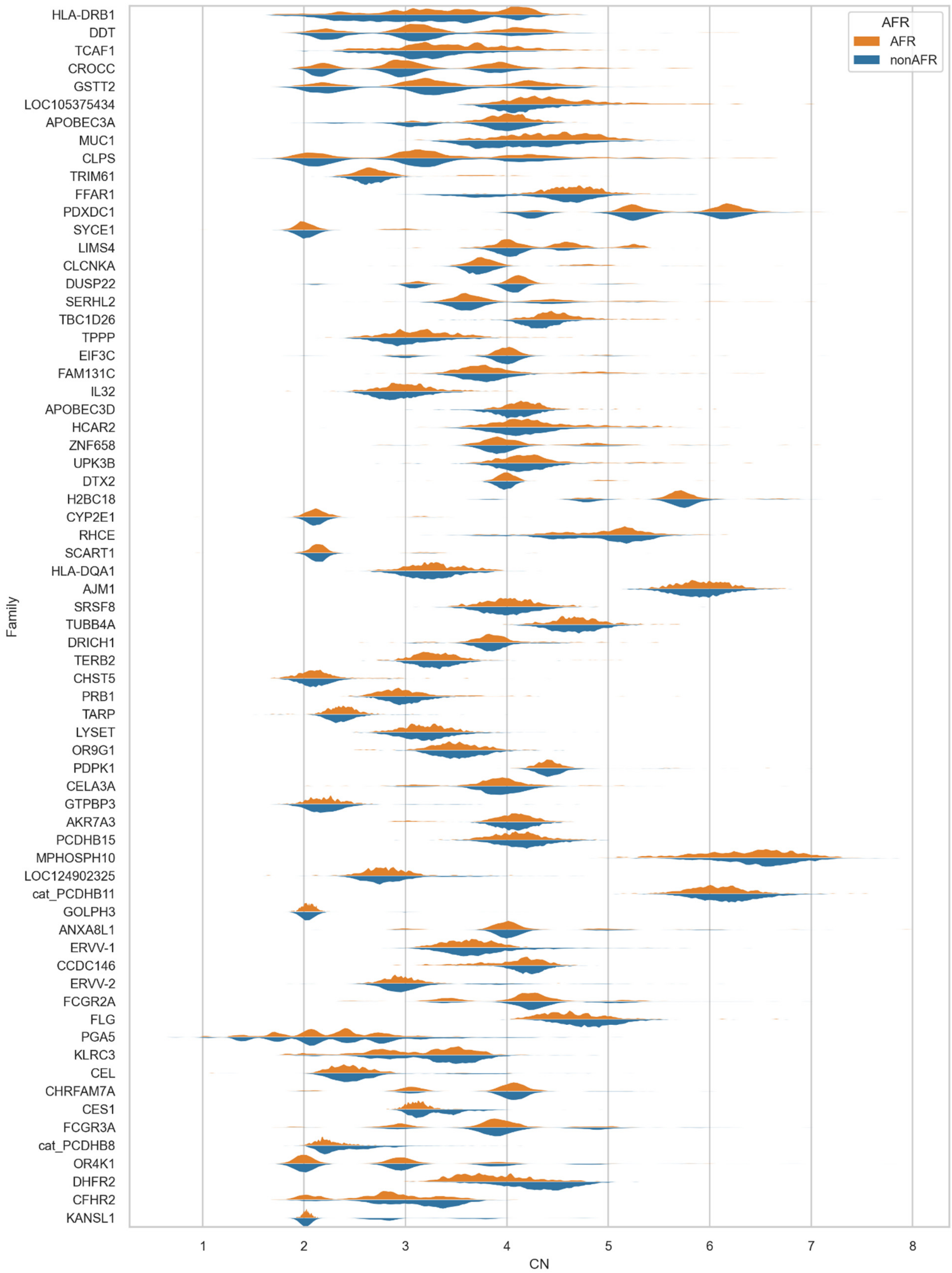
HLA-DRB1
DDT
TCAF1
CROCC
GSTT2
LOC105375434
APOBEC3A
MUC1
CLPS
TRIM61
FFAR1
PDXDC1
SYCE1
LIMS4
CLCNKA
DUSP22
SERHL2
TBC1D26
TPPP
EIF3C
FAM131C
IL32
APOBEC3D
HCAR2
ZNF658
UPK3B
DTX2
H2BC18
CYP2E1
RHCE
SCART1
HLA-DQA1
AJM1
SRSF8
TUBB4A
DRICH1
TERB2
CHST5
PRB1
TARP
LYSET
OR9G1
PDPK1
CELA3A
GTPBP3
AKR7A3
PCDHB15
MPHOSPH10
LOC124902325
cat_PCDHB11
GOLPH3
ANXA8L1
ERVV-1
CCDC146
ERVV-2
FCGR2A
FLG
PGA5
KLRC3
CEL
CHRFAM7A
CES1
FCGR3A
cat_PCDHB8
OR4K1
DHFR2
CFHR2
KANSL1
Family
1
2
3
4
5
6
7
8
CN
AFR
AFR
nonAFR

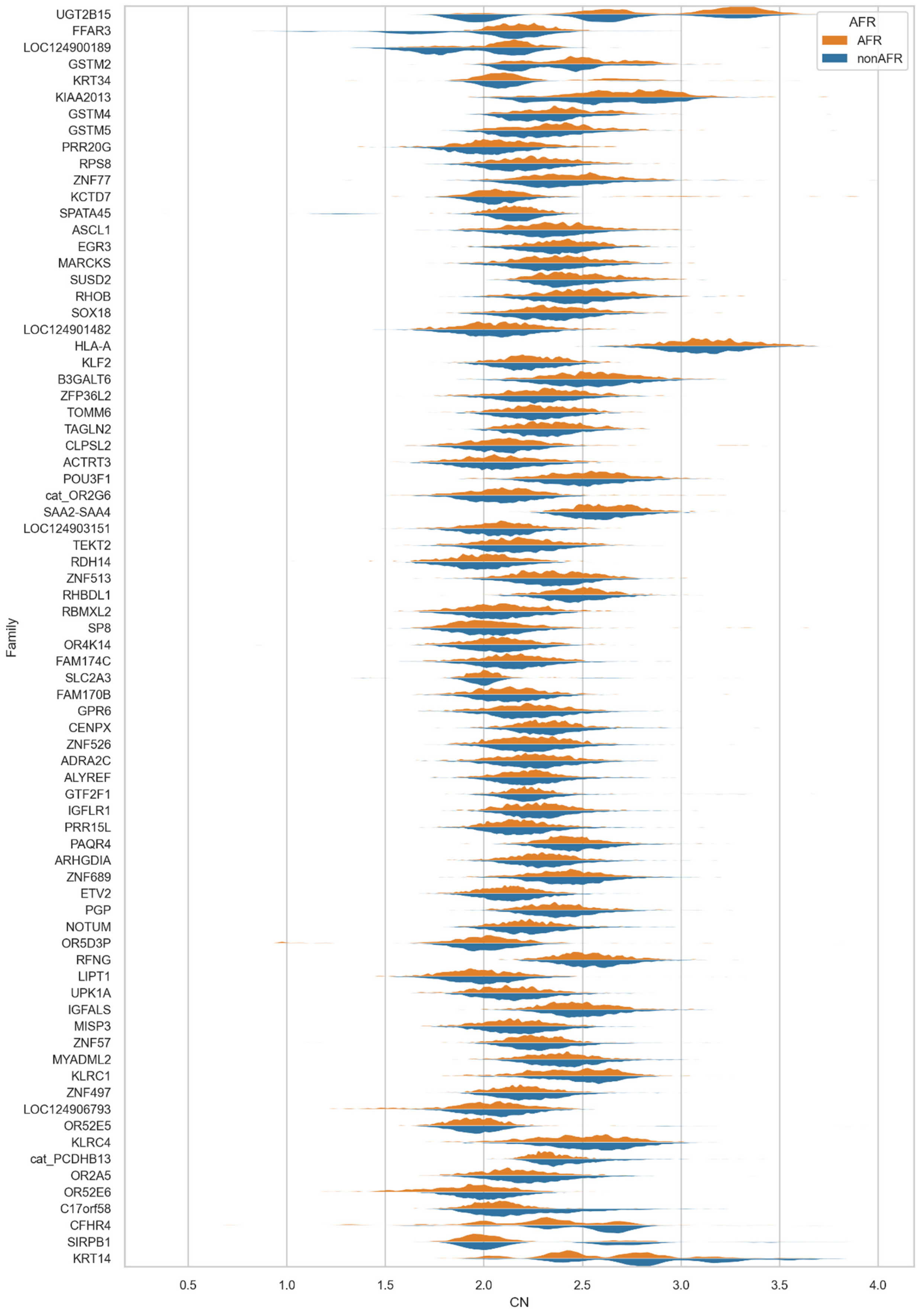
UGT2B15
FFAR3
LOC124900189
GSTM2
KRT34
KIAA2013
GSTM4
GSTM5
PRR20G
RPS8
ZNF77
KCTD7
SPATA45
ASCL1
EGR3
MARCKS
SUSD2
RHOB
SOX18
LOC124901482
HLA-A
KLF2
B3GALT6
ZFP36L2
TOMM6
TAGLN2
CLPSL2
ACTRT3
POU3F1
cat_OR2G6
SAA2-SAA4
LOC124903151
TEKT2
RDH14
ZNF513
RHBDL1
RBMXL2
SP8
OR4K14
FAM174C
SLC2A3
FAM170B
GPR6
CENPX
ZNF526
ADRA2C
ALYREF
GTF2F1
IGFLR1
PRR15L
PAQR4
ARHGDIA
ZNF689
ETV2
PGP
NOTUM
OR5D3P
RFNG
LIPT1
UPK1A
IGFALS
MISP3
ZNF57
MYADML2
KLRC1
ZNF497
LOC124906793
OR52E5
KLRC4
cat_PCDHB13
OR2A5
OR52E6
C17orf58
CFHR4
SIRPB1
KRT14
Family
0.5
1.0
1.5
2.0
2.5
3.0
3.5
4.0
CN
AFR
AFR
nonAFR

Supplementary Figure 7. Population-stratified gene copy number in 1000 Genomes Project. To validate population differences in gene copy number, we repeated the analysis on an unrelated subset of high-coverage Illumina data from the 1000 Genomes Project, excluding samples identified as related by published pedigree or with cryptic relationships (2nd degree or less) as identified by Somalier (Pedersen, Genome Medicine 2020). Individuals from the highly-admixed ACB, ASW, and PUR populations were also excluded, leaving n=2,196.

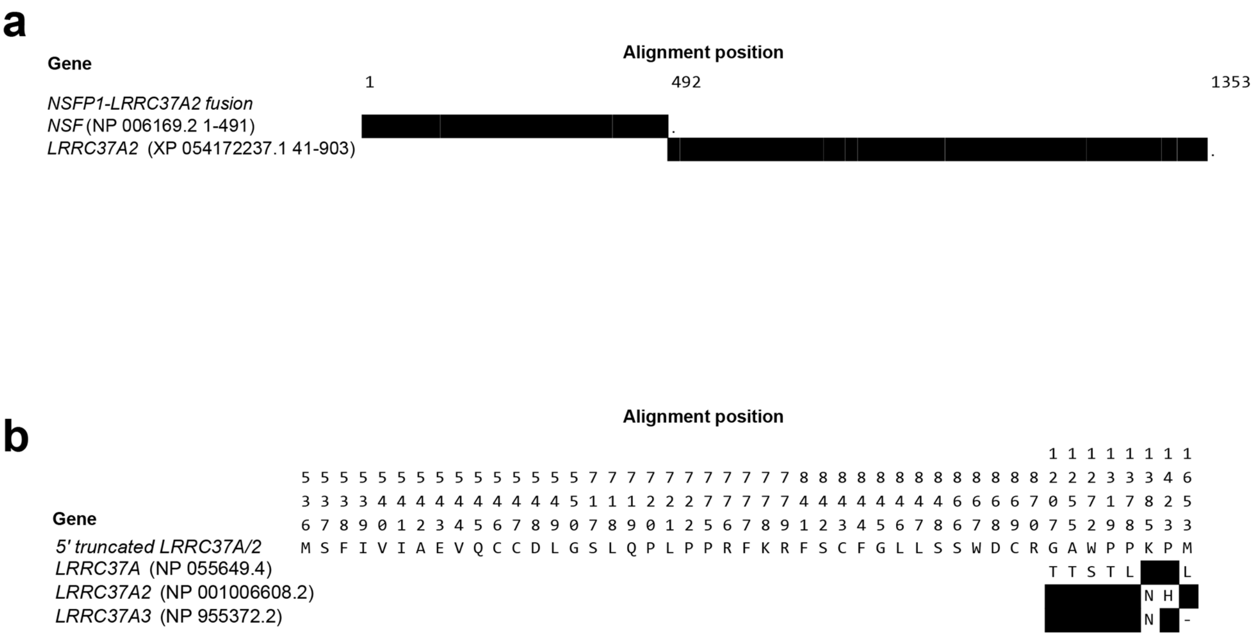

Supplementary Figure 8. Multiple sequence alignment of novel *LRRC37A* family gene predictions compared to reference annotations. Black boxes indicate matches between the novel prediction and reference annotations. **(a)** The *NSFP1-LRRC37A2* gene prediction encodes a fusion between *NSFP1* and *LRRC37A2*. **(b)** The 5' truncated *LRRC37A/2* gene prediction has 39 novel amino acids followed by a hybrid of *LRRC37A* and *LRRC37A2*.

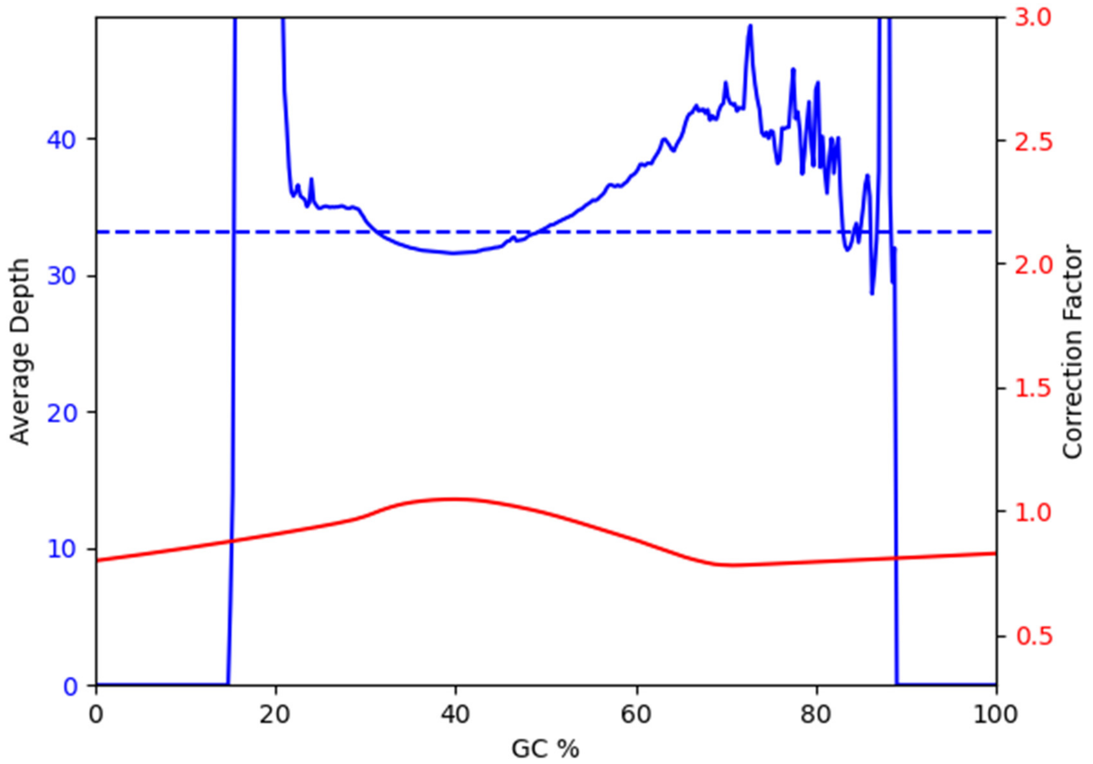

Supplementary Figure 9. Adjustment factor for gene copy number estimation. The read depth of k-mers from decomposed T2T genome assembly is shown as a function of GC composition (blue). Even in a finished genome where there is no experimental or technical error, k-mer read depth is not uniform, since as GC and AT content increases so too does the number of low-complexity k-mers mapping elsewhere. Based on this, we estimated an adjustment factor required by fastCN (red line) to correct for this bias.

SUPPLEMENTARY NOTE

Full list of all consortium members:

Human Genome Structural Variation Consortium (HGSVC)

Adam M. Phillippy[1], Alexander T. Dilthey[2,3], Arda Söylev[2,4], Arvis Sulovari[5], Benedict Paten[6], Bida Gu[7], Carolyn A. Paisie[8], Charles Lee[8], Chen-Shan Chin[9], Chong Li[10,11], Christine R. Beck[8,12], David Porubsky[5], DongAhn Yoo[5], Evan E. Eichler[5,13], Feyza Yilmaz[8], Gianni V. Martino[14,15,16], Glenn Hickey[6], Glennis A. Logsdon[5,17], Haoyu Cheng[18], Heng Li[19,20], Hufsah Ashraf[2,4], Jan O. Korbel[21], Jana Ebler[2,4], Jiaqi Li[22,23], Jonathan Crabtree[24], Katherine M. Munson[5], Keisuke K. Oshima[17], Kendra Hoekzema[5], Keon Rabbani[7], Likhitha Surapaneni[25], Lisbeth A. Guethlein[26], Marc Jan Bonder[27,28], Mark Gerstein[22,23], Mark J.P. Chaisson[7], Mark Loftus[14,15], Matthew Jensen[22,23], Michael C. Zody[29], Mike E. Talkowski[30,31,32], Mikko Rautiainen[33], Mir Henglin[2,4], Miriam K. Konkel[14,15], Nicholas R Pollock[34], Olanrewaju Austine-Orimoloye[25], Parithi Balachandran[8], Patrick Hasenfeld[21], Paul J. Norman[34,35], Peter A. Audano[8], Peter Ebert[2,36], Pille Hallast[8], Qihui Zhu[8,37], Ryan E. Mills[38], Sarah E. Hunt[24], Scott E. Devine[24], Sergey Koren[1], Stephan Scholz[3], Timofey Prodanov[2,4], Tobias Marschall[2,4], Tobias Rausch[21], Vasiliki Tsapalou[21], Weichen Zhou[38], William T. Harvey[5], Xinghua Shi[10,11], Xuefang Zhao[30,31], Ying Zhou[19,20], Youngjun Kwon[5], Yunzhe Jiang[22,23], Yuwei Song[39], Zechen Chong[39]

1. Genome Informatics Section, Center for Genomics and Data Science Research, National Human Genome Research Institute, National Institutes of Health, Bethesda, MD, USA
2. Center for Digital Medicine, Heinrich Heine University, Düsseldorf, Germany
3. Institute of Medical Microbiology and Hospital Hygiene, Medical Faculty, Heinrich Heine University, Düsseldorf, Germany
4. Institute for Medical Biometry and Bioinformatics, Medical Faculty and University Hospital Düsseldorf, Heinrich Heine University, Düsseldorf, Germany
5. Department of Genome Sciences, University of Washington School of Medicine, Seattle, WA, USA
6. UC Santa Cruz Genomics Institute, University of California, Santa Cruz, CA, USA
7. Department of Quantitative and Computational Biology, University of Southern California, Los Angeles, CA, USA
8. The Jackson Laboratory for Genomic Medicine, Farmington, CT, USA
9. Foundation of Biological Data Sciences, Belmont, CA, USA
10. Temple University, Department of Computer and Information Sciences, College of Science and Technology, Philadelphia, PA, USA
11. Temple University, Institute for Genomics and Evolutionary Medicine, Philadelphia, PA, USA
12. The University of Connecticut Health Center, Farmington, CT, USA
13. Howard Hughes Medical Institute, University of Washington, Seattle, WA, USA
14. Clemson University, Department of Genetics & Biochemistry, Clemson, SC, USA
15. Center for Human Genetics, Clemson University, Greenwood, SC, USA
16. Medical University of South Carolina, College of Graduate Studies, Charleston, SC, USA

17. Perelman School of Medicine, University of Pennsylvania, Department of Genetics, Epigenetics Institute, Philadelphia, PA, USA
18. Department of Biomedical Informatics and Data Science, Yale School of Medicine, New Haven, CT, USA
19. Department of Data Science, Dana-Farber Cancer Institute, Boston, MA, USA
20. Department of Biomedical Informatics, Harvard Medical School, Boston, MA, USA
21. European Molecular Biology Laboratory (EMBL), Genome Biology Unit, Heidelberg, Germany
22. Department of Molecular Biophysics and Biochemistry, Yale University, New Haven, CT, USA
23. Program in Computational Biology and Bioinformatics, Yale University, New Haven, CT, USA
24. Institute for Genome Sciences, University of Maryland School of Medicine, Baltimore, MD, USA
25. European Molecular Biology Laboratory, European Bioinformatics Institute, Wellcome Genome Campus, Hinxton, Cambridge, United Kingdom
26. Department of Structural Biology, School of Medicine, Stanford University, Stanford, CA, USA
27. Department of Genetics, University of Groningen, University Medical Center Groningen, Groningen, the Netherlands; Oncode Institute, Utrecht, The Netherlands
28. Division of Computational Genomics and Systems Genetics, German Cancer Research Center, Heidelberg, Germany
29. New York Genome Center, New York, NY, USA
30. Program in Medical and Population Genetics, Broad Institute of MIT and Harvard, Cambridge, MA, USA
31. Center for Genomic Medicine, Massachusetts General Hospital, Boston, MA, USA
32. Department of Neurology, Harvard Medical School, Boston, MA, USA
33. Institute for Molecular Medicine Finland (FIMM), University of Helsinki, Helsinki, Finland
34. Department of Biomedical Informatics, University of Colorado School of Medicine, Aurora, CO, USA
35. Department of Immunology and Microbiology, University of Colorado School of Medicine, Aurora, CO, USA
36. Core Unit Bioinformatics, Medical Faculty and University Hospital Düsseldorf, Heinrich Heine University, Düsseldorf, Germany
37. Stanford Health Care, Palo Alto, CA, USA
38. Department of Computational Medicine & Bioinformatics, University of Michigan, MI, USA
39. Department of Biomedical Informatics and Data Science, Heersink School of Medicine, University of Alabama, Birmingham, AL, USA
