# Supplementary material for: Structural polymorphism and diversity of human segmental duplications
Source: Nat Genet. 2025 Jan 8;57(2):390–401. doi: 10.1038/s41588-024-02051-8 (PMC11821543; doi:10.1038/s41588-024-02051-8)
Supplement: Supplementary file 2 — Reporting Summary [file 41588_2024_2051_MOESM2_ESM.pdf]

nature portfolio

Corresponding author(s): Evan E Eichler

Last updated by author(s): Nov 18, 2024

Reporting Summary

Nature Portfolio wishes to improve the reproducibility of the work that we publish. This form provides structure for consistency and transparency in reporting. For further information on Nature Portfolio policies, see our Editorial Policies and the Editorial Policy Checklist.

Statistics

For all statistical analyses, confirm that the following items are present in the figure legend, table legend, main text, or Methods section.

n/a	Confirmed	
☐	☒	The exact sample size (*n*) for each experimental group/condition, given as a discrete number and unit of measurement
☐	☒	A statement on whether measurements were taken from distinct samples or whether the same sample was measured repeatedly
☐	☒	The statistical test(s) used AND whether they are one- or two-sided<br>*Only common tests should be described solely by name; describe more complex techniques in the Methods section.*
☒	☐	A description of all covariates tested
☐	☒	A description of any assumptions or corrections, such as tests of normality and adjustment for multiple comparisons
☐	☒	A full description of the statistical parameters including central tendency (e.g. means) or other basic estimates (e.g. regression coefficient) AND variation (e.g. standard deviation) or associated estimates of uncertainty (e.g. confidence intervals)
☐	☒	For null hypothesis testing, the test statistic (e.g. *F*, *t*, *r*) with confidence intervals, effect sizes, degrees of freedom and *P* value noted<br>*Give P values as exact values whenever suitable.*
☒	☐	For Bayesian analysis, information on the choice of priors and Markov chain Monte Carlo settings
☐	☒	For hierarchical and complex designs, identification of the appropriate level for tests and full reporting of outcomes
☒	☐	Estimates of effect sizes (e.g. Cohen's *d*, Pearson's *r*), indicating how they were calculated

Our web collection on statistics for biologists contains articles on many of the points above.

Software and code

Policy information about availability of computer code

Data collection	No software was used to collect data.
Data analysis	PacBio HiFi data were processed with hifiasm (v0.16), minimap2 (v2.24), RagTag (v2.1), RepeatMasker (v4.1), BCFtools (v1.9). PacBio Iso-Seq data were processed with minimap2 (v2.24), Liftoff (v1.6.3), SQANTI3 (v5.2), GeneMark (v4.3), BLAST (v.2.15).<br>The code for processing PacBio HiFi data is available at Github (https://github.com/hrrsjeong/pangenome_SD) and Zonodo (https://doi.org/10.5281/zenodo.11623075).

For manuscripts utilizing custom algorithms or software that are central to the research but not yet described in published literature, software must be made available to editors and reviewers. We strongly encourage code deposition in a community repository (e.g. GitHub). See the Nature Portfolio guidelines for submitting code & software for further information.

Data

Policy information about availability of data

All manuscripts must include a data availability statement. This statement should provide the following information, where applicable:

- Accession codes, unique identifiers, or web links for publicly available datasets
- A description of any restrictions on data availability
- For clinical datasets or third party data, please ensure that the statement adheres to our policy

The raw sequencing data generated in this study are available under project ID PRJEB58376 (https://www.ebi.ac.uk/ena/browser/view/PRJEB58376) and the HPRC

year 1 PacBio HiFi data are available under PRJNA730823 (https://ncbi.nlm.nih.gov/bioproject/PRJNA730823). HPRC genome assemblies are available online (https://github.com/human-pangenomics/HPP_Year1_Assemblies). HGSVC genome assemblies used in this study are available online (https://eichlerlab.gs.washington.edu/public/HPRC_HGSVC_assemblies/). The raw genome sequencing data generated from this study are available online (https://ftp.1000genomes.ebi.ac.uk/vol1/ftp/data_collections/HGSVC3/). The T2T-CHM13 (v.2.0) reference genome used in this study is available under PRJNA559484 (https://www.ncbi.nlm.nih.gov/bioproject/PRJNA559484).

Research involving human participants, their data, or biological material

Policy information about studies with human participants or human data. See also policy information about sex, gender (identity/presentation), and sexual orientation and race, ethnicity and racism.

Reporting on sex and gender	N/A
Reporting on race, ethnicity, or other socially relevant groupings	We report analyses of publicly available human genome sequencing data generated by the 1000 Genomes Project (1KG; https:// www.internationalgenome.org/home) and their associated genetic ancestry information, as established and described by the 1000 Genomes Project (https://www.internationalgenome.org/category/population/).
Population characteristics	see above
Recruitment	see above
Ethics oversight	see above

Note that full information on the approval of the study protocol must also be provided in the manuscript.

Field-specific reporting

Please select the one below that is the best fit for your research. If you are not sure, read the appropriate sections before making your selection.

☒ Life sciences ☐ Behavioural & social sciences ☐ Ecological, evolutionary & environmental sciences

For a reference copy of the document with all sections, see nature.com/documents/nr-reporting-summary-flat.pdf

Life sciences study design

All studies must disclose on these points even when the disclosure is negative.

Sample size	We generated HiFi sequence data from 53 human (14 trio and 39 non-trio) samples. We also analyzed whole-genome assemblies from diverse humans generated by the Human Pangenome Reference Consortium (HPRC). This included 47 trio binning assemblies. In total, we analyzed 170 independent genome assemblies from 85 human specimens representing 38 African and 47 non-African samples. The sample size is sufficient to compare segmental duplication content.
Data exclusions	No data were excluded from the analyses.
Replication	Independent biological replicates were used for genome assembly as indicated in the manuscript.
Randomization	Randomization is not applicable to this study because we did not perform any experiments with treatment or control groups that would necessitate randomization between the subjects.
Blinding	Blinding is not applicable to this study because we did not perform any experiments with treatment or control groups that would necessitate blinding.

Reporting for specific materials, systems and methods

We require information from authors about some types of materials, experimental systems and methods used in many studies. Here, indicate whether each material, system or method listed is relevant to your study. If you are not sure if a list item applies to your research, read the appropriate section before selecting a response.

Materials & experimental systems

n/a	Involved in the study
☒	☐ Antibodies
☐	☒ Eukaryotic cell lines
☒	☐ Palaeontology and archaeology
☒	☐ Animals and other organisms
☒	☐ Clinical data
☒	☐ Dual use research of concern
☒	☐ Plants

Methods

n/a	Involved in the study
☒	☐ ChIP-seq
☒	☐ Flow cytometry
☒	☐ MRI-based neuroimaging

Eukaryotic cell lines

Policy information about cell lines and Sex and Gender in Research

Cell line source(s)	Lymphoblastoid cell lines used for 1KG collection were obtained from the NHGRI Sample Repository at the Coriell Institute for Medical Research.
Authentication	Genomic variants in the sequencing data were compared to previously published data.
Mycoplasma contamination	All cell lines are negative for mycoplasma contamination.
Commonly misidentified lines (See ICLAC register)	No commonly misidentified cell lines were used in this study.

Plants

Seed stocks	N/A
Novel plant genotypes	N/A
Authentication	N/A
